# Supplementary material for: Plant Peroxisome-Targeting Effector MoPtep1 Is Required for the Virulence of Magnaporthe oryzae
Source: Int J Mol Sci. 2022 Feb 24;23(5):2515. doi: 10.3390/ijms23052515 (PMC8910473; doi:10.3390/ijms23052515)
Supplement: Supplementary file 1 [file ijms-23-02515-s001.zip › ijms-1531093-supplementary.pdf]

Supplemental Table S1. Primers used in this study

Primer name	Sequence (5'-3')
**Secretory validation vector construction primers**	
pSUC2-MoPtep1-FL-F	CGGAATTTTAATTAAGAATTCATGCGGTCTATAGCTATCCTGA
pSUC2-MoPtep1-FL-R	CACTATAGGGAGAACCTCGAGGCTGGGAGGGGGAACGGCTTG
pSUC2-MoPtep1-NS-F	CGGAATTTTAATTAAGAATTCATGCTTCAGATCCCAGACCTCC
**Knockout mutant strains construction primers**	
HPT-F	CTTGGCTGGAGCTAGTGGAGGT
HPT-R	CCCGGTCGGCATCTACTCTATTC
HPT-F1	CGTTGCAAGACCTGCCTGAA
HPT-R1	GGATGCCTCCGCTCGAAGTA
HPT-LBCK	GACAGACGTCGCGGTGAGTT
HPT-RBCK	TCTGGACCGATGGCTGTGTAG
MoPtep1-LBCK	ACAGTGCTTGGGAATGGGAC
MoPtep 1-LB-R	ACCTCCACTAGCTCCAGCCAAGGATGAAATCATGGCTAGATT
MoPtep 1-RBCK	CTCATCCAGCGTCAAGGCCA
MoPtep 1-RB-F	GAATAGAGTAGATGCCGACCGGGGAAGATTTGGATGCAGCAAC
MoPtep 1-LB-F	TCGAGTCGTTCTCCAAGTTT
MoPtep 1-RB-R	TTCACCACTCCGACAGGTAC
**Knockout mutant strain checking primers**	
MoPtep1-F	ATGCGGTCTATAGCTATCCT
MoPtep1-R	TTAGCTGGGAGGGGGAACGG
**Complementary strains construction primers**	
C-MoPtep1-pGTN-F-1	GGGAACAAAAGCTGGGTACCGAGAAGCTCGTGGCCGTGTC
C-MoPtep1-pGTN-R	ATTCTAGAACTAGTGGATCCGCTGGGAGGGGGAACGGCTT
**Complementary strains checking primers**	
pGTN-cx-F	CATTAGGCAC CCCAGGCTTT
pGTN-cx-R	TCGTGACCA CCCTGACCTAC
***MoPtep1* expression primers**	

RT-MoPtep1-qF	TCAGATCCCAGACCTCCTG
RT-MoPtep1-qR	CGTGACTGTATGGTTGTTTG
RT-Mo-Actin-qF	TGACGTCCGAAAGGATCTGT
RT-Mo-Actin-qR	CCTGCTTCGAGATCCACATC
**Subcellular localization and cell death observation primers**	
MoPtep1-FL-GFP-F	CGCTCTAGAACTAGTGGATCCATGCGGTCTATAGCTATCCT
MoPtep1-FL-GFP-R	GGGCCCCCCCTCGAGGTCGACGCTGGGAGGGGGAACGGCTT
MoPtep1-NS-GFP-F	CGCTCTAGAACTAGTGGATCCATGCTTCAGATCCCAGACCT

Supplemental Table S2. Information on candidate interacting proteins of MoPtep1

Name	Number of amino acids/aa	GenBank accession	Gene description	Extend information
**HZ-7**	270	XP_015646760.1	**momilactone A synthase-like**	Momilactone: An antibacterial compound found in husks of rice, a natural defense substance that has the activity of suppressing the growth of pathogens such as blast fungus.
**IP-4**	177	XP_015618577.1	**thaumatin-like protein**	This family is also referred to as pathogenesis-related group 5 (PR5), as many thaumatin-like proteins accumulate in plants in response to infection by a pathogen and possess antifungal activity.
**HZ-4**	152	XP_040380872.1	**plastocyanin, chloroplastic**	Plastocyanin; Plastocyanin is a type I copper protein and functions in the electron transfer from PSII to PSI.

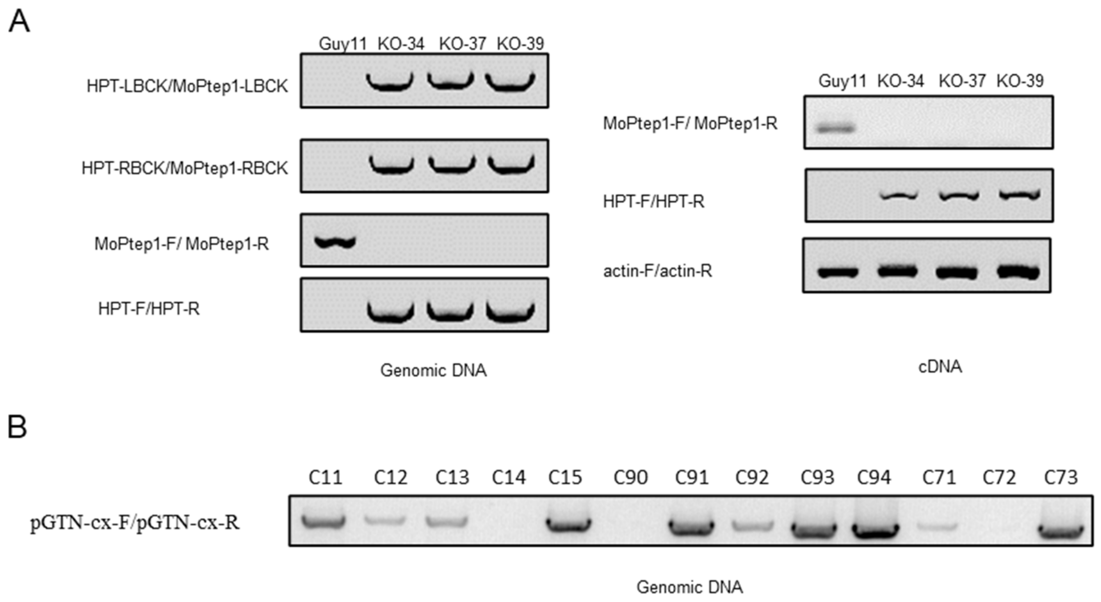

Supplemental Figure S1: Identification of the transformants using PCR method. (A) identification of *MoPtep1* ko mutant strains via PCR method. Use the Split-PCR method to knock out the *MoPtep1* gene. Using wild-type strains and transformants' genomic DNA as templates, primers were used to amplify corresponding bands to verify transformants. At the same time, RT-PCR was used for testing. MoPtep1-F/ MoPtep1-R cannot amplify bands were the correct knockout strains. (B) identification of the complementary strain of MoPtep1 via PCR method. Use vector pGTN to construct *MoPtep1* complementary vector, transform the constructed vector into the protoplasts of *MoPtep1* ko mutant strains, and screen with G418-resistant plates, Use pGTN-cx-F/pGTN-cx-R for PCR screening of the grown transformants, and amplify the corresponding bands of the positive transformants.
